# Relationships Among Bullying Experiences, Mood Symptoms and Suicidality in Subjects with and Without Autism Spectrum Conditions

**DOI:** 10.3390/brainsci15101114

**Published:** 2025-10-16

**Authors:** Liliana Dell’Osso, Benedetta Nardi, Stefano Pini, Gabriele Massimetti, Lucrezia Castellani, Francesca Parri, Filippo Del Grande, Chiara Bonelli, Carmen Concerto, Matteo Di Vincenzo, Bianca Della Rocca, Maria Salvina Signorelli, Laura Fusar-Poli, Camilla Figini, Pierluigi Politi, Eugenio Aguglia, Mario Luciano, Barbara Carpita

**Affiliations:** 1 Department of Clinical and Experimental Medicine, University of Pisa, 56126 Pisa, Italy; liliana.dellosso@unipi.it (L.D.); stefano.pini@unipi.it (S.P.); gabriele.massimetti@unipi.it (G.M.); castellanilucrezia91@gmail.com (L.C.); francyparri@icloud.com (F.P.); f.delgrande1@studenti.unipi.it (F.D.G.); chiarabonelli.95@hotmail.it (C.B.); barbara.carpita@unipi.it (B.C.); 2 Department of Clinical and Experimental Medicine, Institute of Psychiatry, University of Catania, 95124 Catania, Italy; c.concerto@policlinico.unict.it (C.C.); maria.signorelli@unict.it (M.S.S.); eugenio.aguglia@unict.it (E.A.); 3 Department of Psychiatry, University of Campania “L. Vanvitelli”, 80138 Naples, Italy; dr.matteodivincenzo@gmail.com (M.D.V.); dellarocca.bianca@gmail.com (B.D.R.); mario.luciano@unicampania.it (M.L.); 4 Department of Brain and Behavioral Sciences, University of Pavia, 56126 Pavia, Italy; laura.fusarpoli@unipv.it (L.F.-P.); camilla.figini@unipv.it (C.F.); pierluigi.politi@unipv.it (P.P.)

**Keywords:** bullying, victimization, peer victimization, autism spectrum disorder, autistic traits, suicidality, mood symptoms

## Abstract

**Background**: Bullying is a major public health issue with long-term psychological consequences, particularly for individuals with Autism Spectrum Disorder (ASD) or subthreshold autistic traits, known as “broad autistic phenotype” (BAP). Prior studies have suggested increased vulnerability to victimization and mood disorders in these populations, but the association between bullying, autistic traits, and affective symptoms remains underexplored. **Methods**: A total of 98 individuals with at least one ASD symptom (BAP group) and 159 healthy controls (HCs) were recruited. Participants were classified into four groups based on ASD symptoms and bullying history. Standardized self-report instruments (AdAS Spectrum, AQ, MOODS-SR) assessed autistic traits, mood symptoms, and suicidality. Group comparisons, correlation analyses, and multiple regression models were conducted to evaluate the relationships between bullying, autistic traits, and mood disturbances. **Results**: BAP individuals, particularly those with ASD, reported significantly higher rates of bullying than HCs. Bullied BAP participants exhibited the highest burden of mood symptoms and suicidality. Regression analyses identified both autistic traits and bullying history as significant predictors of suicidal ideation and overall suicidality, though only autistic traits predicted suicidal behaviors. AQ and MOODS-SR scores were positively correlated, especially in depressive and rhythmicity domains. **Conclusions**: Autistic traits and bullying experiences independently and interactively contribute to increased mood symptomatology and suicidality. These findings underscore the importance of early identification and targeted support for at-risk individuals with ASD or BAP, particularly those with a history of victimization.

## 1. Introduction

Bullying is a widespread phenomenon, defined as a persistent and intentional pattern of aggressive or harmful behavior towards people who are seen as weaker and unable to defend themselves [1]. Bullying, which is defined as recurrent victimization in a relationship of power imbalance—where bullies gain power while victims lose their own,—can take many different forms, occur frequently, and involve varying degrees of aggressiveness, from name-calling and teasing to verbal, physical, and social abuse, both in person and via online platforms [2,3]. Recent studies estimate that up to 24% and, among children and adolescents with neurodevelopmental and psychiatric conditions, the prevalence can rise up to 42% [4]. Bullying rates among children show notable regional variation, with reported prevalence rates of 22.8% in Central America, 25% in Europe, 31.7% in North America, and 48.2% in Sub-Saharan Africa [5]. For adolescents, a meta-analysis of 80 studies examining various bullying subtypes found that 36% experience traditional bullying, while 15% are affected by cyberbullying [6]. Due to its high prevalence and significant impact on health, social, and educational outcomes during childhood and adolescence, the World Health Organization (WHO) has recognized childhood bullying as a serious public health issue [7]. While the public health consequences of bullying have been acknowledged for some time, it is only in the past decade that research has focused on prospective studies exploring the long-term effects of bullying into adulthood. Being the target of bullying exposes victims to negative consequences in multiple areas, including relational, psychological, physical, and overall health [8]. Victims are at an increased risk of experiencing adverse psychosocial outcomes, such as low self-esteem, feelings of helplessness, social skill deficits, and peer rejection [6]. They often struggle academically, facing difficulties with concentration, absenteeism, and poor grades [1]. In the long term, these individuals may have trouble forming lasting relationships and adapting to adult roles [9]. According to Låftman et al., victims of bullying and cyberbullying are more likely to develop a pessimistic outlook on the future, experience a reduced quality of life, and suffer from negative health outcomes [10]. Additionally, sleep disturbances are common, often due to intrusive thoughts about bullying during the night [11]. In this framework, the onset of mental health issues has been strongly linked to bullying victimization, with research suggesting a graded association [6], with studies reporting a doubled risk for victimized children to develop major depression later in life, compared to their non-victimized peers [11]. Victims of bullying tend to have a poorer perception of their overall health and are more likely to experience internalizing disorders such as psychosomatic complaints, anxiety, depression, and suicidal thoughts [9,12,13,14]. Ultimately, a growing body of evidence now confirms that being bullied during childhood or adolescence can contribute to the development of mental health issues later in life, such as depression, anxiety, and suicidal tendencies [15]. As a result, the global public health issue of childhood bullying has gained increasing attention.

Bullying is usually targeted at children and adolescents with vulnerabilities, such as those who differ from their peer group, belong to a minority group, or have disabilities [8]. In particular, a population at high risk of bullying and distressing social experiences is that of subjects belonging to the autism spectrum. Autism Spectrum Disorder (ASD) is defined as a neurodevelopmental condition characterized by impaired communication and interaction in various social contexts, restricted interests, repetitive behaviors or activities, including the possibility of having peculiar responses to sensory stimuli such as hyper- or hyporeactivity [16]. ASD can vary along a continuum of manifestations and the presence of intellectual or linguistic impairment increases the severity of the condition. Recently, the scientific community has recognized the importance of paying attention not only to the overt forms of the disorder, but also to the more nuanced and subthreshold ones, which were initially identified in first-degree relatives of subjects with ASD, and subsequently extended to the general population, and known as “broad autistic phenotype” (BAP) [17]. The BAP includes all those autistic-like characteristics such as inflexibility, tendency to isolation, detached personality and strong and narrow interests, which, due to number or severity, do not meet the diagnostic criteria for a diagnosis of full-blown ASD [18]. The importance of identifying these subthreshold autistic traits lies in the fact that they seem to exert a significantly negative impact on quality of life, also being a vulnerability factor for the development of other psychopathological and somatic conditions [17,18]. To date, research remains limited on the specific experiences of interpersonal violence in individuals with ASD, but existing studies suggest a higher risk for child maltreatment, bullying, and sexual victimization [19,20,21]. In fact, youth with ASD or elevated autistic traits are estimated to be 40–94% more likely to experience bullying than their typically developing peers [22,23]. For children with ASD, the likelihood of suffering physical, emotional, or sexual abuse is greater compared to children without disabilities [24,25,26,27,28]. Particularly, deficits in socio-communicative skills may serve as key risk factors for both victimization and perpetration of violence in adults with ASD [29,30]. Individuals with ASD often struggle with social reasoning, think literally, and may fail to recognize contextual cues, all of which could contribute to an increased vulnerability to bullying [28,31,32]. Different types of bullying, including verbal, physical, relational, and cyberbullying, have been shown to exert differential impacts on individuals with neurodevelopmental conditions. Each of these types of bullying may exert different psychological effects on victims. Previous literature has shown that individuals with ASD are not only more vulnerable to direct forms of aggression but are also disproportionately affected by relational and cyber forms, which may be more difficult to detect by caregivers and educators. In particular, individuals with ASD are more vulnerable not only to overt physical or verbal aggression but also to subtle relational forms of victimization, which may exacerbate social difficulties and emotional dysregulation. This heightened vulnerability is often related to the socio-communicative difficulties, literal interpretation of social cues, and reduced coping resources typically associated with ASD, which can exacerbate the negative outcomes of victimization. Importantly, these experiences may further interact with autistic traits, contributing to increased risk for mood disturbances and suicidality [19,21,23,27].

Therefore, considering evidence that both autistic traits and victimization increase the risk of developing affective disorders later in life, and given that individuals with autistic traits are more vulnerable both to bullying and to trauma- and stress-related symptoms, we aimed to investigate the relationship between bullying experiences, mood symptoms, and autistic traits in individuals with autistic traits compared to healthy controls (HCs). The study specifically explores how the presence of previous experiences of bullying may be associated with increased mood spectrum symptoms, including suicidality, when associated or not with the presence of autistic traits.

## 2. Materials and Methods

### 2.1. Subjects and Procedures

For the purpose of the study, we recruited a total sample of 257 subjects. Participants were classified into three groups: (1) 34 individuals with a clinical diagnosis of ASD (ASD group), confirmed by a qualified clinician according to DSM-5 criteria; (2) 64 individuals without an ASD diagnosis but endorsing at least one autistic symptom as defined by DSM-5 criteria on the AdAS Spectrum (BAP1 group, representing subthreshold autistic traits); and (3) 158 healthy controls (HC), without an ASD diagnosis and without clinically relevant autistic traits. The recruitment was performed in the University Psychiatric Clinics of Pisa, Napoli, Pavia, Catania. BAP1 subjects were recruited among students of three Italian university of excellence (Scuola Superiore S.Anna in Pisa, Collegio Universitario di Merito in Pavia, Scuola Superiore in Catania). Individuals under 18, those with significant intellectual or language impairments, defined as an IQ < 70 or the presence of a documented clinical diagnosis of intellectual disability, schizophrenia, a history of substance abuse, major neurological or medical conditions, or those unable to provide written informed consent or complete psychometric questionnaires independently were excluded. Detailed information about the study was provided, and participants had the chance to ask questions before signing the written informed consent.

The final sample was made of 98 subjects with at least one criterium for ASD (BAP) and 158 HCs. Among BAP subjects, 34 reported a clinical ASD diagnosis (ASD group) and 64 met only one symptomatologic criterium for BAP (BAP1).

The sample was divided in four groups based on the diagnostic category and the presence of a history of bullying, investigated with the AdAS Spectrum item number 19. Specifically, subjects who showed symptoms of at least one criterion for ASD diagnosis and reported a history of bullying were included in the bullied BAP group, while those who responded negatively to AdAS Spectrum number 19 were included in the BAP group. Similarly, HCs who responded positively to AdAS Spectrum item 19 were included in the bullied HC group, whereas those who did not were included in the HC group. The division into four groups (BAP bullied, BAP non-bullied, HC bullied, HC non-bullied) was designed to highlight the interactive effects of autistic traits and bullying history.

**Figure 1:**
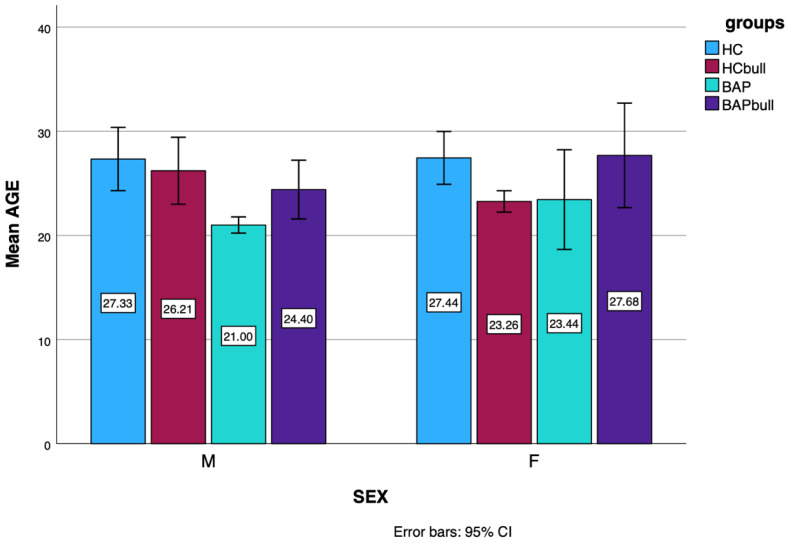
Barr chart: age and gender distribution.

From such division resulted four groups:

- 39 BAP individuals who did not experience bullying (F:16; M:23; mean age: 25.46 ± 9.40);
- 59 BAP individuals who experienced bullying (Bullied BAP) (F:19; M:40; mean age: 22.00 ± 5.93);
- 112 HCs who experienced bullying (Bullied HCs) (F:27; M:19; mean age: 27.35 ± 10.22);
- and 46 HCs who did not experience bullying (F:70; M:42; mean age: 24.48 ± 4.99) (see Figure 1).

We acknowledge that the “bullying” condition may represent distinct experiential contexts across groups, given that autistic traits themselves confer an intrinsic vulnerability to negative social experiences.

The study followed the ethical guidelines outlined in the Declaration of Helsinki and was approved by the ethical committee of Azienda Ospedaliera Universitaria Pisana (AOUP).

### 2.2. Measures

#### 2.2.1. The Adult Autism Spectrum (AdAS Spectrum)

The AdAS Spectrum is a 160-item self-report questionnaire designed to assess a wide variety of autism-related characteristics in people without cognitive or linguistic problems. It is divided into seven categories, each with a distinct focus: *Childhood and Adolescence*, *Verbal Communication*, *Nonverbal Communication*, *Empathy*, *Inflexibility and Adherence to Routine*, *Restricted Interests and Rumination*, and *Hyper- and Hyporeactivity to Sensory Input*. A validation study found that the instrument has good internal consistency, great Test–Retest reliability, and strong convergent validity with existing dimensional measures of autism [33].

A positive response to item 19 of the AdAS spectrum (“were you ever teased by school-mates or bullied?”) was used to discriminate subjects who had suffered bullying episodes from those who had not.

#### 2.2.2. The Autism-Spectrum Quotient (AQ)

The AQ is a commonly used questionnaire designed to offer a self-report assessment of autistic features for individuals with normal Intelligence Quotients (IQ). It has 50 questions that test five different areas: *Social Skills*, *Attention Switching*, *Attention to Detail*, *Communication*, and *Imagination*. The AQ’s Test–Retest and inter-rater reliability were strong, and items in each of the five domains had moderate to high alpha values, indicating reasonable construct validity [34].

#### 2.2.3. The MOOD Spectrum—Self Report (MOODS-SR)

The MOODS-SR is a comprehensive evaluation instrument used to evaluate states of depression, mania, and hypomania. It comprises symptoms, behaviors, and lifestyle choices associated with various levels of mood dysregulation, including severe and moderate affective disorders. It has 160 items separated into seven areas that measure *Manic Mood*, *Manic Energy*, and *Manic Cognition*, as well as *Depressed Mood*, *Depressive Energy*, and *Depressive Cognition*. Another domain focuses on *Rhythmicity* and vegetative processes, which include feeding, sleeping cycles, and sexual behavior [35]. As per in previous studies, MOODS-SR items 102 to 107 have been utilized to assess the presence of suicidal thought and suicidal conduct [36,37,38,39,40].

### 2.3. Statistical Analysis

Prior to recruitment, we calculated the sample size to ensure a power of at least 80%, considering four diagnostic groups compared and a difference in clinical relevance, calculated as the residual standard deviation between the group scores, equal to 1. A sample size of 39 subjects for each group (equal to the smallest group), and therefore a non-centrality parameter of Phi = 2.208, guarantees, for an α = 0.05, a power of 97.6%.

A chi-square analysis was performed in order to compare the presence of bullying experiences among groups.

Subsequently, a one-way ANOVA analysis followed by an appropriated post-hoc analysis, was used to compare score obtained at the MOODS-SR and AQ total and domains scores between groups.

Pearson correlation analysis was then performed in the bullied group (BAP and HC) in order to investigate the presence of significant correlation between MOOD-SR and AQ domains scores.

Three linear regression analyses were then performed using, respectively, overall suicidality, suicidal ideation and suicidal behavior as the dependent variables and AQ total score and being bullied as independent variable, in order to investigate the presence of a statistical predictor of suicidality.

Student *t*-test were then used to compare MOOD-SR and AQ domains and total scores among subjects who showed only one criterion for the diagnosis of ASD (BAP) who have or have not experienced bullying.

Another student *t*-test analysis was subsequently performed to compare MOOD-SR and AQ domains and total scores among subjects who showed at least two clinical symptoms of ASD who have or have not experienced bullying.

All statistical analyses were performed with SPSS version 26.0.

## 3. Results

Performing a chi-square analysis, bullying experiences were significantly more represented in BAPs than in HCs (60.2% of BAPs reported bullying experiences vs. 29.11% of HCs; chi-square = 24.165, *p* = 0015). Moreover, among patients with ASD diagnosis (N = 34), 27 reported bullying experiences (79.41%) while only 32 on 68 subjects with BAP1 reported them (50%), highlighting a significantly higher presence of victimization in ASD subjects also when comparted to subjects with subthreshold autistic traits. The latter, however, still reported a significantly higher presence of victimization with respect to HCs (29.11%; chi-square = 32.105; *p* < 0.001).

Results from the one-way ANOVA analysis showed that BAP subjects who experienced bullying scored significantly higher in all AQ and MOOD-SR domains as well as in MOOD-SR suicidality compared to HCs (see Table 1). Similarly, bullied BAPs scored higher on the MOODS-SR total than the other groups, while bullied BAPs and HCs scored higher than HCs and did not differ from each other.

**Figure 2:**
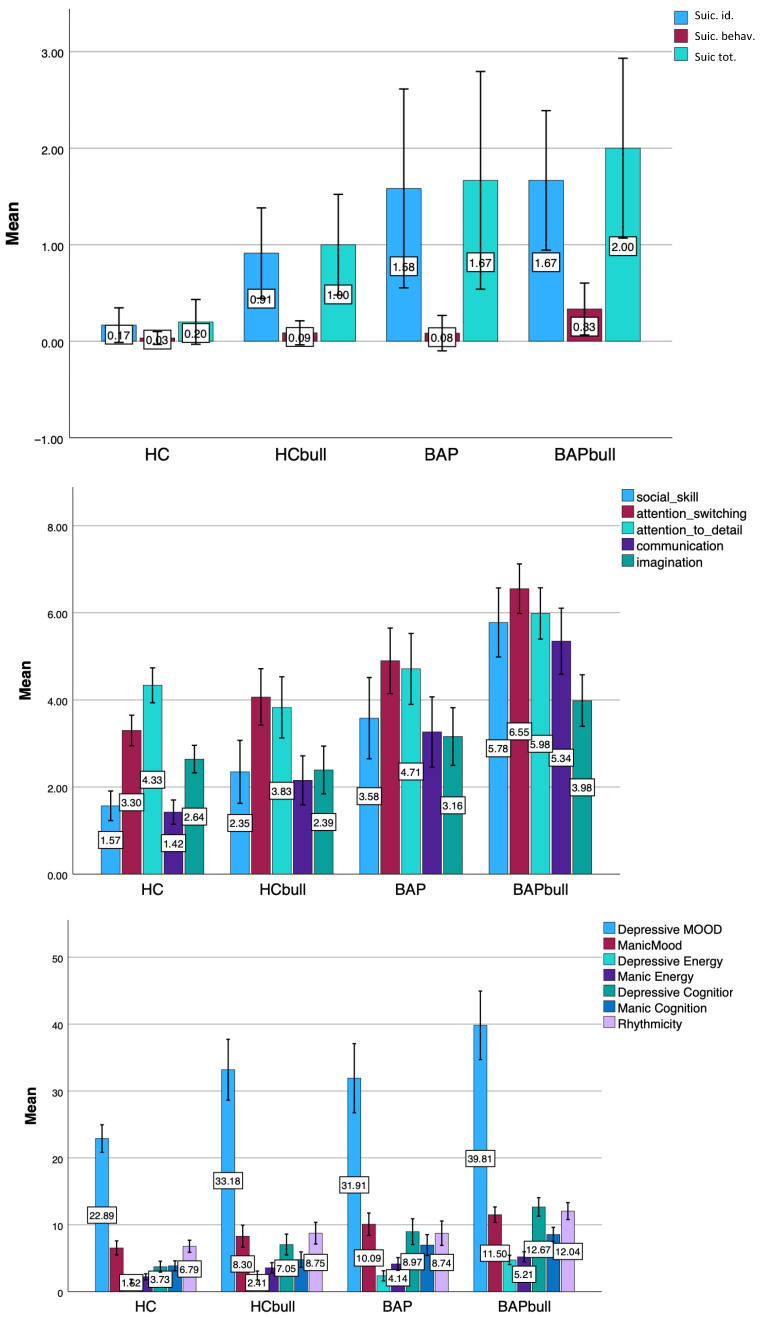
Bar chart of the comparison of MOOD-SR and AQ scores between groups.

Moreover, bullied BAP scored higher than HCs and bullied HCs in MOOD-SR *Suicidal ideation*, *Manic* *mood* and *Manic cognition* domain, while in turn non-bullied BAP scored higher than HCs. On the other hand, for MOOD-SR *Suicidal behaviors* the only significant difference was found between bullied BAP and HCs, while regarding the overall suicidality all BAP subjects (bullied and non) scored significantly higher than HCs. Furthermore, bullied BAP scored significantly higher than the other three groups in AQ *Social skills*, *Attention switching*, *Attention to detail*, and *Communication* domains and AQ total score as well as in MOOD-SR *Depressive energy*, *Depressive Cognition* and *Rhythmicity* domains. Similarly, BAP scored higher than HCs in AQ *Social skills*, *Attention switching*, and *Communication* domains as well as in MOOD-SR *Depressive energy* domain, and higher than both HCs and bullied HCs in AQ total score. BAP and bullied HCs also scored higher than HCs in MOOD-SR *Depressive mood*, *Depressive cognition* and *Manic energy* domain (Figure 2).

Results from the Pearson’s correlation analysis showed significant positive correlations between all AQ domains and total score and MOOD-SR *Depressive mood*, and *Depressive cognition* domains and total score (see Table 2).

MOOD-SR *Depressive energy* domain positively correlated with all AQ domain and total score with the only exception for AQ *Attention to detail* domain, whereas MOOD-SR *Rhythmicity* domain positively correlated with all AQ domains and total with the only exception of AQ *Imagination* domain. Similarly, MOOD-SR *Manic cognition* domain positively correlated with all AQ domain and total score except for AQ *Social skill* and *Imagination* domains. MOOD-SR *Manic energy* domain positively correlated with AQ *Attention switching* and *Communication* domains scores as well as with its total.

Overall suicidality, and suicidal ideation and behaviors emerged to be positively correlated with AQ *Social skill* and *Attention to detail* domains and total score, the latter being also positively correlated with AQ *Imaging* domain.

Results from the first linear regression analysis performed using overall suicidality as the dependent variable and AQ total score and being bullied as independent variable showed that both variables were statistically predictive of suicidality (see Table 3).

Similarly, results from the second regression performed with the same independent variables and suicidal ideation as dependent variables showed both AQ total score and being bullied were positive predictors of suicidal ideation (see Table 4).

Lastly, results from the third regression performed with the same independent variables and suicidal behavior as dependent variables showed only AQ total score was a positive predictor of suicidal behavior (see Table 5).

We then compared BAP1 subjects that did or did not report a history of bullying. Specifically, 32 subjects belonged to the BAP1 group (mean age: 20.59 ± 1.39) and 32 subjects belonged to the bullied BAP1 group (mean age: 21.50 ± 2.72). The two groups did not significantly differ by age.

**Figure 3:**
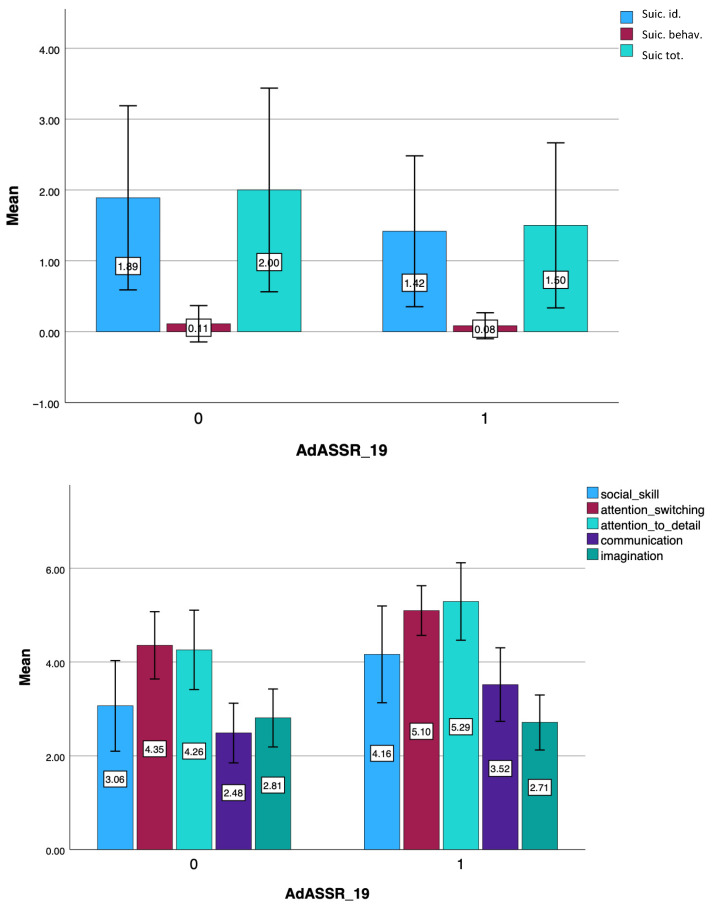

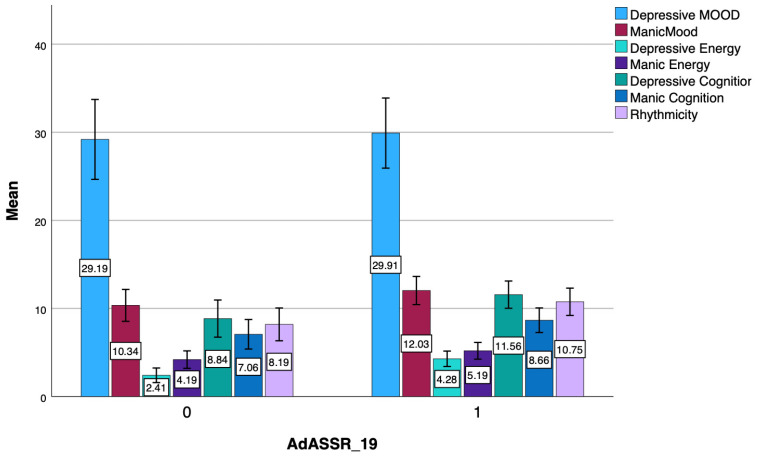
Bar chart of the comparison of MOOD-SR and AQ scores between subjects who showed only one criterion for the diagnosis of ASD who have or have not experienced bullying.

Results from the student *t*-test highlighted that individuals belonging to the bullied BAP group scored significantly higher in AQ *Communication* domain and total score, in MOOD-SR *Depressive Energy*, *Depressive Cognition* and *Rhythmicity* domains compared to BAP1 who were not bullied (see Table 6 and Figure 3).

Lastly, we compared subjects with at least two clinical symptoms of ASD that did or did not report a history of bullying. In particular, 7 subjects belonged to the non-bullied ASD group (mean age: 28.43 ± 12.41) and 27 to the bullied ASD group (mean age: 30.15 ± 12.21).

**Figure 4:**
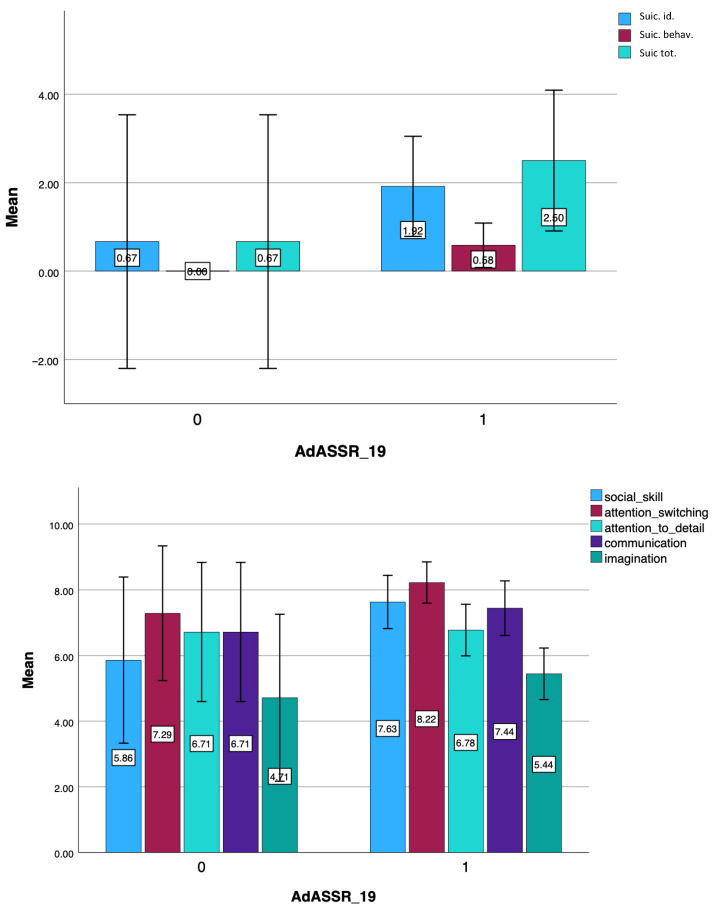

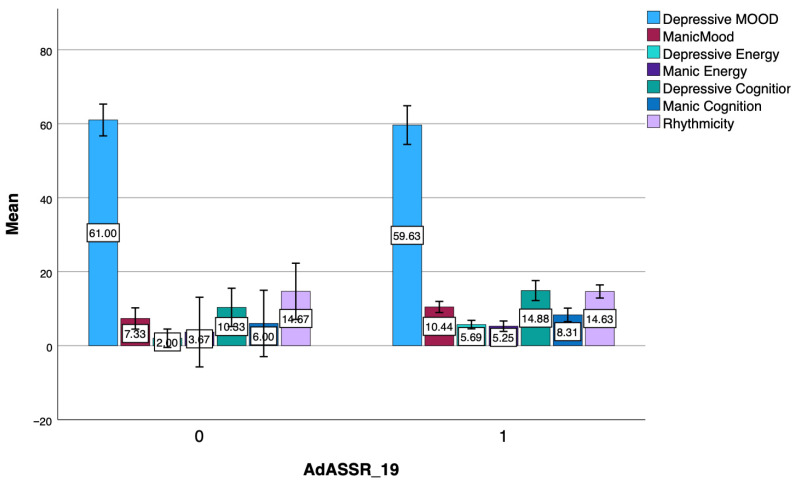
Bar chart comparison of MOOD-SR and AQ scores between subjects with a clinical diagnosis of ASD who have or have not experienced bullying.

Results from the student *t*-test highlighted that subjects belonging to the bullied ASD group scored significantly higher in MOOD-SR *Manic mood* and *Depressive energy* domain, as well as in the suicidal behavior score, compared to non-bullied ASD (see Table 7 and Figure 4).

## 4. Discussion

In this study, we aimed to investigate the relationship between bullying experiences, mood symptoms and autistic traits in individuals on the autistic spectrum.

Our findings highlighted that a higher proportion of BAP subjects reported bullying experiences with respect to HCs, with ASD reporting bullying experiences in an even higher proportion than BAP1. This is in line with previous literature, which highlighted how ASD subjects, but also people with subthreshold autistic traits are more exposed to the risk of peer victimization [41].

Moreover, individuals in the BAP group who reported experiences of bullying exhibited significantly.

Moreover, individuals in the BAP group who reported experiences of bullying exhibited significantly higher levels of global affective symptoms compared to the other groups, as shown by the total MOODS-SR score. On the other hand, BAP subjects and victimized HCs also showed higher total MOODS-SR scores than HCs, although lower than those of victimized BAPs. This suggests that both the aspect of individual vulnerability associated with the autism spectrum and the presence of victimization may be associated with a greater burden of mood spectrum symptoms, which is at its highest when the two factors occur together. Recent research has provided substantial evidence on the role of stress experienced in early childhood as a major risk factor, especially in vulnerable subjects, for the onset and persistence of mental disorders [42,43,44]. Regarding the scores reported for the individual domains of the MOODS-SR, victimized BAP subjects still reported significantly higher scores than HCs. Furthermore, most mood symptoms were still more present in BAP subjects, in line with the greater vulnerability of these subjects [17]. Furthermore, some mood symptoms appear to be more present than controls even in subjects who have suffered bullying without autistic traits, such as depressive mood, depressive cognition and manic energy, which could therefore be specifically exacerbated by bullying experiences even in the absence of psychopathological vulnerabilities.

In this framework, early exposure to stressful events has been shown to sensitize the central nervous system, leading to disruptions in the circuits that regulate emotions and stress, which in turn may contribute to the development of mood and anxiety disorders [45,46]. Many studies also suggested that the circuits involved in the stress response, along with their associated neurotransmitters, become hypersensitive in individuals who experience repeated traumatic events during childhood. This heightened reactivity to stress can persist into adulthood, increasing vulnerability to the disorders mentioned above [45,47,48]. Thus, adverse experiences during critical developmental periods may create long-lasting vulnerabilities to stress, predisposing individuals to a broad range of mental and physical health issues that can either emerge or worsen under acute or chronic stress.

Moreover, individuals with BAP who experienced bullying scored higher than those in the other three groups in the MOOD-SR domains of *Depressive Energy*, *Depressive Cognition*, and *Rhythmicity*. Additionally, both BAP and bullied HC participants scored higher in the *Depressive Cognition*, *Depressive Mood* and *Manic Energy* domains compared to the HC group, indicating that both autistic traits and traumatic experiences may play a role in increasing vulnerability to depressive symptoms, but also on the emergence of counter-polar mood dysregulation [49]. Similarly, regardless of bullying experience, individuals with BAP scored higher in the *Depressive Energy* domain on the MOOD-SR compared to both bullied and non-bullied HCs. When comparing BAP to bullied BAP individuals, our results revealed that those who were bullied showed more pronounced depressive symptoms, particularly in terms of energy, cognition, and rhythmicity. This suggests that autistic traits may independently contribute to these specific psychopathological dimensions, while the experience of bullying further exacerbates these effects. Furthermore, a wide range of environmental adversities may contribute to depressive vulnerability. While specific stressors such as bullying, abuse, or family loss have been consistently linked to increased risk of depression, socioeconomic adversity, including poverty, also represents an important factor. Recent studies confirm that financial strain and low socioeconomic status are associated with higher rates of depressive symptoms across the lifespan [50]. These findings underscore that multiple forms of adversity, both social and economic, may interact with autistic traits and bullying experiences in shaping mental health outcomes. The link between childhood trauma and depression has long been investigated. A systematic review of the literature carried in 2023 reveals how in children subjected to traumatic events there is a functional alteration of the anterior cingulate cortex and the precuneus, alterations that are also detected in subjects affected by major depression and chronic pain [51]. Furthermore, alterations in the reward systems, which are involved in the development of anhedonia, have also been detected in subjects who had suffered maltreatment in childhood [51,52,53]. A 2019 meta-analysis also demonstrated how the presence of early stressful life events was not only a generic risk factor for the development of depression during life, but specifically increased the possibility of developing it even before the age of 18 [54]. Furthermore, various types of stressful events were compared with each other, demonstrating that not all had the same weight: for example, poverty was not related to an increase in the risk of depression, while physical and psychological abuse (such as bullying), death of a family member and domestic violence were positively correlated [53]. Although research on the potential connection between childhood victimization and disruptions in circadian rhythms remains limited, some studies have highlighted the impact of early trauma on systems involved in stress regulation, such as the hypothalamic-pituitary-adrenal (HPA) axis [46,47,55]. Specifically, acute and chronic stress, such as peer victimization, has been linked to HPA axis activation and the subsequent release of cortisol, which normally follows a circadian rhythm [56]. However, prolonged activation of the HPA axis can become maladaptive, leading to delayed stress response shutdown or inadequate cortisol responses, which over time can significantly impair both physical and mental health [57,58]. Recent studies have found that peer victimization is associated with disruptions in HPA axis activity, including lower morning cortisol levels [58,59,60] and a blunted cortisol response to stress [61,62,63]. Additionally, research on monozygotic twins suggests that this down-regulation of the HPA axis is not solely due to genetic factors [64]. The relationship between peer victimization and reduced HPA axis activity likely reflects the cumulative effects of repeated HPA axis activation, which ultimately leads to alterations in cortisol secretion and disrupts the physiological circadian rhythm [55,65].

Regardless of the presence of victimization, patients with only BAP showed higher scores in most domains related to the presence of mood disorders. The association between autism spectrum and symptoms related to mood disorders is also confirmed by the correlation analysis, which reports significant correlations between the AQ and most of the domains of the MOODS-SR, as well as between their totals, although with a higher correlation with symptoms related to the depressive sphere. The link between affective disorders and autistic traits has been recognized for some time. It is well-established that individuals with ASD often have comorbid psychiatric conditions, which can significantly affect their social and academic functioning [66]. In particular, adults with ASD are at heightened risk for mental health disorders, especially anxiety and depression [67,68]. However, estimates of comorbid mental health disorders in this population vary considerably. Some studies report anxiety or depression rates as high as 70% [69,70], while others report much lower rates, with depression found in fewer than 1% of cases [71]. Clinical studies focusing on treatment-seeking adults, however, suggest that depression is common in those with ASD, with rates ranging from 20% to 35% [70,72], compared to around 7% in the general population [73].

In parallel, the presence of autistic traits in individuals with psychiatric disorders has been increasingly recognized [42,43,44,74,75]. For example, a 2008 study found that 38% of patients with major depressive disorder scored above the clinical threshold for autistic traits [76]. Additional research in adults has further supported the link between autistic-like traits and psychiatric conditions like depression and anxiety [77]. The finding that adults with major depressive disorder exhibit higher levels of AT than the general population suggests a potential pathophysiological overlap between ASD and other psychiatric disorders. Although AT are continuously distributed in the general population, they appear in greater frequency in high-risk groups, particularly clinical samples of psychiatric patients [17]. Although the finding that bullied BAP individuals reported the highest levels of mood symptoms may seem expected, this empirical evidence is important to confirm the additive impact of individual and environmental vulnerabilities. Moreover, our analyses highlighted that the burden in bullied BAPs was not only global but also characterized by specific domains, such as depressive energy, depressive cognition, and rhythmicity, suggesting distinct psychopathological patterns. Finally, regression analyses revealed differential predictive roles, with both autistic traits and bullying history associated with suicidal ideation, but only autistic traits predicting suicidal behavior. These findings provide a more nuanced understanding of risk mechanisms beyond the simple observation of higher overall scores.

Interestingly, while bullied BAP scored higher than controls, bullied and non, in MOOD-SR *Manic mood* and *Manic cognition* domains, significant difference was also observed between non-bullied BAP individuals and HCs. This is in line with the association of autism spectrum not only with major depression but also with bipolar disorder [78]. Furthermore, it is believed that the diagnostic rate may be underestimated as in subjects on the autistic spectrum, bipolar disorder it often presents in an atypical manner compared to the general population [78]. Also, the link between mania, bipolar disorder and traumatic events in childhood has been widely demonstrated and confirmed. Indeed, it is believed that approximately 30–50% of subjects with bipolar disorder have suffered trauma of various kinds in the first years of their life, including peer victimization, bullying and maltreatment [79].

Similarly, when comparing bullied and non-bullied ASD subjects, those who reported a history for bullying scored significantly higher in MOOD-SR *Manic mood* and *Depressive energy* domain. These evidences were confirmed by the results of the correlation analysis that showed a positive correlation between depressive and manic dimension and autistic traits. Such data support the previously discussed comorbidity between bipolar disorder and autistic traits. Furthermore, this data also highlights the close link between autism and catatonia, as the presence of expansive mood associated with very low levels of energy appear similar to the oscillations between stupor and agitation found in very severe forms of catatonia. Indeed, a recent meta-analysis reported how at least 10.4% of autistic patients met the criteria for catatonia, and how autistic subjects with catatonia showed greater severity of motor symptoms [80]. In this framework, it has been hypothesized the existence of an autistic-catatonic continuum in which catatonic manifestation may represent transitory periods of extreme severity of the autistic symptoms [38,81,82]. Moreover, catatonia, a complex psychomotor syndrome, has been linked to trauma since its origins, so much so that one of its first manifestations was called shell-shock because it was found in soldiers who survived explosions in the First World War [83]. Precisely because of its intrinsic link to trauma, some authors have suggested that catatonia may stem as a pathological response to serious traumatic events, like the freeze-fight-flight response commonly found in animals that in situations of danger [84,85].

Furthermore, bullied BAP scored significantly higher than the other three groups in AQ total score and *Social skills*, *Attention switching*, *Attention to detail*, and *Communication* domains. In particular, even when comparing exclusively BAP and bullied BAP, our results showed that bullied individuals had greater impairments in *Communication*. Our data that reduced social skills and communication difficulties might be associated with a greater predisposition to be exposed to traumatic events of interpersonal nature, including bullying. Recent studies have indeed proposed that communication difficulties in children may play a significant role in social challenges and deficits, serving as a potential risk factor for peer victimization [86,87]. Children with impairments in communication or deficits in social skills often struggle to connect with their peers, particularly during childhood and adolescence [86,88,89]. These children tend to have fewer friendships, lower peer acceptance, and are at a higher risk of being bullied or victimized [89,90,91]. In turn, bullying can negatively impact the social interactions of victims, leading to social rejection, exclusion, and further victimization [92]. Several studies have found that children and adolescents with communication and social difficulties are up to three times more likely to be bullied compared to their typically developing peers [91,93,94,95]. However, other research has not supported these findings [96], indicating that further studies are needed to fully understand this relationship. On the other hand, while attention switching deficits and attention to details are peculiar characteristics of BAP subjects [21,97,98], on the other traumatic experiences have been suggested to promote an enhancement of the brain circuits involved in increased attention to detail and rumination, reducing attention switching abilities and ultimately worsening the symptomatology [99]. Indeed, individuals with a history of bullying seem to develop greater cognitive rigidity and increased difficulty in diverting their attention from distressing thoughts or memories. Individuals who have been victims of bullying may therefore develop ruminative thinking about past events, making it difficult to shift their attention to more positive or neutral thoughts, as their mind is stuck in the past [100].

Furthermore, BAP, bullied and non-bullied, reported higher overall suicidal tendencies than only non-victimized HC, while victimized HC obtained intermediate scores that were not different between the other groups. Regarding suicidal ideation, victimized BAP reported higher scores than victimized and non-victimized HC, while non-victimized BAP reported higher scores only than non-victimized HC. Instead, regarding suicidal behaviors, only victimized BAP seemed to differ significantly from HC. These results seem to confirm a role of both autistic traits and victimization on suicidal risk, with a higher risk when the two components of individual vulnerability and environmental factors are associated. Moreover, results provided by the regression analysis that showed how both autistic traits and a history of bullying were statistically predictive of overall suicidality and suicidal ideation, while concerning suicidal behavior, the presence of autistic traits emerged as the only statistical predictor, thus revealing the greater association with the autistic spectrum as a vulnerability factor for the implementation of suicidal actions. Similarly, when comparing bullied and non-bullied ASD subjects, those who reported a history for bullying scored significantly higher in in the suicidal behavior score, while there was no difference for the BAP group. This data suggests an important role of the severity of autistic traits as a factor that, combined with interpersonal victimization, can favor the development of suicidal behavior. In accordance with current literature, our results demonstrate how autistic traits represent vulnerability factors towards suicidal behaviors and thoughts [101,102] and that, although to a lesser extent early traumatic events such as peer victimization, are also associated with a higher tendencies towards suicidal behaviors, especially when they occur in a context of individual vulnerability [103,104]. Research has consistently shown that individuals with ASD face a significantly higher risk of suicidality [74,105,106,107,108]. However, an increasing body of studies suggests that even subthreshold autistic traits—traits that do not meet the full criteria for ASD—can contribute to suicidality risk in both clinical and non-clinical populations [108,109]. Many studies have found a connection between autistic traits and suicidal thoughts or behaviors [105,106,107,108,109], as well as a greater susceptibility to negative life events, impaired social problem-solving skills, and depression [110,111,112]. The ongoing discourse about the relationship between autistic traits and suicidal tendencies has led to the identification of several key factors that may explain this association. For instance, traits commonly seen in individuals on the autism spectrum, such as perfectionism, neuroticism, and introversion, are strongly linked to an increased risk of suicide [113,114,115]. Additionally, autistic traits are often associated with social challenges like isolation, bullying, and the pressure to hide or “mask” these traits to fit in with neurotypical peers [110,116,117,118]. Recent research highlights that autistic traits themselves serve as independent risk factors for suicide attempts, regardless of other co-occurring conditions [101,119]. Being a victim of bullying has been shown to be associated with internalizing disorders such as depression and anxiety [120,121,122,123]. Several systematic reviews and meta-analyses have shown that any level of involvement in bullying (either as a victim, a bully or both) is associated with suicidal ideation [103,104]. Specifically, in their 2008 review that included 27 studies conducted in general population samples of children and adolescents, the authors concluded that all bullying subtypes were associated with suicidal ideation and that the strongest association was found for bully-victims [124]. One meta-analysis focused on victimization and suicidal ideation, and confirmed that bullying victimization was associated with an increased risk for suicidal ideation [125]. In a meta-analysis that included 47 studies, each bullying subtype was found to be associated with suicidal ideation [126].

Lastly, overall suicidality and suicidal ideation and behaviors emerged to be positively correlated with AQ *Social skill* and *Attention to detail* domains and total score, the latter being also positively correlated with AQ Imagination domain. Such results support the hypothesis that suicidal ideation is correlated with deficits in social reciprocity. Social interactions are crucial for emotional support, validation, and a sense of belonging. When someone struggles with these skills, they may experience social isolation, which is a major risk factor for both depression and suicidality. Feeling disconnected from others can create a sense of hopelessness, making the individual more vulnerable to suicidal thoughts. Similarly, a greater attention to details, characteristic of individuals with cognitive rigidity, predisposes to a mixed ruminative tendency in a perfectionist key, which is presumed to be a facilitating factor for non-conservative ideation and suicidal behaviors [127,128]. Lastly, although the literature focusing on such association is still scant, it is possible that deficits in imagination may be correlated with suicidality for several reasons, particularly in how they affect an individual’s ability to envision future possibilities, cope with adversity, or find alternative solutions to problems [129,130]. In this framework, imagination is a vital tool for developing adaptive coping strategies. People who struggle with imaginative thinking may have difficulty coming up with new ways to handle stress, anxiety, or difficult emotions and instead fall into rigid patterns of thinking, where they feel stuck in their current state of emotional distress [131].

### Limitations

The present study must be considered in light of several limitations. For instance, it is important to acknowledge that mood symptoms and suicidality may be influenced by multiple factors other than bullying, including comorbid psychiatric conditions. The current study did not control for all potential confounders, and therefore, the associations observed between bullying, autistic traits, and affective symptoms should be interpreted with caution. Then, the limited sample size may reduce statistical power and limit the generalizability of the findings. Another limit is that bullying was assessed with a single item rather than through validated multi-item instruments. While this approach has been employed in prior studies in regards of different variables, it prevents a detailed characterization of bullying experiences. Another limitation concerns the recruitment strategy, as BAP participants were mainly recruited from universities of excellence, while ASD participants were recruited from psychiatric clinics. This difference may reflect not only distinct clinical conditions but also systematic differences in socioeconomic, educational, or psychosocial background, which could have influenced the results. Future studies should consider more homogeneous recruitment strategies to minimize potential selection bias and improve the comparability between groups. Furthermore, differences between age and sex in the groups could have influenced the results. In addition, the cross-sectional nature of the study prevents inferences on causal or temporal relationships between the variables involved. Moreover, the lack of data regarding variables such as education, comorbidities, and symptom severity limits interpretation of group differences, and no adjustment was made for potential confounding variables above, which may have biased the observed associations. In addition, while a single-item measure cannot capture the full complexity of bullying experiences, our study primarily aimed to examine the association between bullying history (as reported by participants) and mood symptoms and autistic traits, rather than to provide a detailed typology of bullying itself. Another limitation concerns the psychometric instruments adopted. While the MOODS-SR and AQ scales allow for a comprehensive assessment of mood and autistic traits, they also capture a wide range of affective and cognitive features that may not be exclusively related to bullying or suicidality. This could have introduced additional variability into the results. Another limitation is the absence of systematic data regarding participants’ pharmacological or psychotherapeutic treatments. Finally, the use of self-report questionnaires could facilitate the presence of bias in the form of over- or underestimation of symptoms by patients.

## 5. Conclusions

Our results showed that subjects with autism spectrum disorder who had been bullied during childhood/adolescence showed a significantly higher global burden of mood disorder symptoms compared to all other groups examined. Furthermore, subjects with autism spectrum disorder who had not been bullied and healthy controls who had been bullied also showed more mood spectrum symptoms compared to healthy controls who had not been victimized. This suggests that both autistic traits and bullying experience in developmental age can be associated with the presence of mood disorders, with a maximum burden when the two factors are concomitant. The study also reported that autistic traits and bullying history were significant predictors of suicidality, with a more severe presence of autism spectrum disorder specifically associated with a greater presence of suicidal behaviors.

## Figures and Tables

**Table 1:** Comparison of MOOD-SR and AQ scores between groups.

	BAP (Mean ± SD)	Bullied BAP (Mean ± SD)	HC (Mean ± SD)	Bullied HC (Mean ± SD)	F	*p*	H^2^	C.I. 95%	
								Lower Bound	Upper Bound
MOOD-SR Suicidality									
Suic. id.	1.37 ± 1.59	1.93 ± 1.65	0.22 ± 0.77	0.81 ± 1.17	17.396	<0.001 *	0.257	0.135	0.354
Suic. behav.	0.08 ± 0.29	0.32 ± 0.63	0.03 ± 0.26	0.12 ± 0.34	3.435	0.019 °	0.080	0.002	0.168
Suic. tot.	1.67 ± 1.77	2.00 ± 2.21	0.20 ± 0.90	1.00 ± 1.21	11.111	<0.001 ^	0.225	0.090	0.333
AQ									
Social Skill	3.61 ± 2.81	5.71 ± 3.03	1.55 ± 1.80	2.35 ± 2.43	40.367	<0.001 ^#^	0.325	0.229	0.401
Att. Switc.	4.87 ± 2.26	6.58 ± 2.16	3.25 ± 1.88	4.06 ± 2.17	34.810	<0.001 ^#^	0.291	0.197	0.369
Att. detail	4.74 ± 2.45	6.02 ± 2.24	4.31 ± 2.11	3.83 ± 2.36	10.324	<0.001 ^§^	0.109	0.041	0.176
Comm.	3.26 ± 2.46	5.30 ± 2.88	1.39 ± 1.47	2.15 ± 1.90	47.097	<0.001 ^#^	0.358	0.263	0.433
Imagin.	3.15 ± 1.99	3.98 ± 2.24	2.61 ± 1.69	2.39 ± 1.84	8.383	<0.001 ^¥^	0.091	0.028	0.155
AQ tot.	19.60 ± 8.94	27.64 ± 9.73	13.26 ± 5.11	14.78 ± 7.26	49.805	<0.001 ^£^	0.375	0.279	0.450
MOOD-SR									
Dep. Mood	31.91 ± 15.04	39.81 ± 17.65	22.89 ± 11.08	33.18 ± 14.96	18.648	<0.001 ^$^	0.192	0.103	0.270
Man. Mood	10.09 ± 4.88	11.50 ± 4.01	6.54 ± 5.63	8.30 ± 5.40	11.753	<0.001 *	0.130	0.054	0.203
Dep. En.	2.37 ± 2.21	4.75 ± 2.41	1.52 ± 1.83	2.41 ± 2.22	26.845	<0.001 ^#^	0.254	0.158	0.335
Man. En.	4.14 ± 2.79	5.21 ± 2.61	2.22 ± 2.44	3.57 ± 2.54	17.204	<0.001 ^$^	0.179	0.092	0.257
Dep. Cogn.	8.97 ± 5.62	12.67 ± 4.78	3.73 ± 4.38	7.05 ± 5.15	41.644	<0.001 ^&^	0.346	0.247	0.424
Man. Cogn.	6.97 ± 4.53	8.54 ± 3.70	3.87 ± 4.01	4.80 ± 3.79	17.612	<0.001 *	0.183	0.095	0.261
Rhythm.	8.74 ± 5.31	12.04 ± 4.37	6.79 ± 4.84	8.75 ± 5.33	12.935	<0.001 ^§^	0.141	0.062	0.216
MOOD-SR tot.	70.37 ± 29.35	94.45 ± 26.60	47.57 ± 26.10	68.05 ± 28.69	35.244	<0.001 ^†^	0.313	0.214	0.392

BAP: individuals without an ASD diagnosis but endorsing at least one autistic symptom; HC: healthy controls; AQ: Autism Quotient; MOOD-SR: MOOD Spectrum-Self report; * Bullied BAP > HC, Bullied HC & BAP > HC; ° Bullied BAP > HC; ^ Bullied BAP, BAP > HC; ^#^ Bullied BAP > BAP, Bullied HC, HC & BAP > HC; ^§^ Bullied BAP > BAP, Bullied HC, HC; ^¥^ Bullied BAP > Bullied HC, HC; ^£^ Bullied BAP > BAP > Bullied HC, HC; ^$^ Bullied BAP, BAP, Bullied HC > HC; ^&^ Bullied BAP > BAP, Bullied HC, HC & BAP, Bullied HC > HC; ^†^: bullied BAP > BAP, bullied HC > HC; significant for *p* < 0.05.

**Table 2:** Pearson’s correlation coefficients between MOOD-SR, AQ and suicidality in the bullied sample (BAP and HCs).

	Social Skill	Att. Switch.	Att. to Detail	Comm.	Imag.	AQ tot. Score
**Dep. Mood**	0.585 **	0.637 **	0.276 **	0.515 **	0.355 **	0.609 **
*p*	<0.001	<0.001	0.008	<0.001	0.001	<0.001
**Manic Mood**	0.072	0.179	0.134	0.101	−0.047	0.112
*c*	0.493	0.089	0.203	0.338	0.661	0.293
**Dep. Energy**	0.450 **	0.475 *	0.198	0.375 **	0.263 *	0.454 **
*p*	<0.001	<0.001	0.058	<0.001	0.012	<0.001
**Manic Energy**	0.146	0.333 **	0.186	0.270 **	0.090	0.259 *
*p*	0.164	0.001	0.075	0.009	0.397	0.013
**Dep. Cognition**	0.486 **	0.487 **	0.350 **	0.481 **	0.293 **	0.538 **
*p*	<0.001	<0.001	0.001	<0.001	0.005	<0.001
**Manic Cognition**	0.128	0.339 **	0.283 **	0.253 *	0.124	0.281 **
*p*	0.223	0.001	0.006	0.015	0.242	0.007
**Rhythmicity**	0.254 *	0.402 **	0.210 *	0.303 **	0.187	0.343 *
*p*	0.015	<0.001	0.044	0.003	0.075	0.001
**MOOD-SR** **tot. score**	0.531 **	0.649 **	0.343 **	0.526 **	0.318 **	0.606 **
*p*	<0.001	<0.001	0.001	<0.001	0.002	<0.001
**Suicidal ideation**	0.327 **	0.249	0.372 **	0.210	0.204	0.337 **
*p*	0.010	0.051	0.003	0.101	0.115	0.008
**Suicidal behav.**	0.357 *	0.262	0.298 *	0.261	0.343 *	0.373 *
*p*	0.012	0.069	0.038	0.071	0.016	0.008
**Suicidality total**	0.355 *	0.185	0.323 *	0.249	0.195	0.327 *
*p*	0.014	0.212	0.027	0.091	0.189	0.025

AQ: Autism Quotient; MOOD-SR: MOOD Spectrum-Self report; * significant for *p* < 0.05, ** significant for *p* < 0.01. Darker blue correspond to stronger correlations; red indicates negative correlation.

**Table 3:** Linear regression analysis performed using suicidality as the dependent variable and AQ total score and being bullied as independent variable.

	B (S.E.)	Beta	t	*p*
constant	−0.246 (0.284)		−0.869	0.387
AQ total score	0.047 (0.015)	0.286	3.131	0.02
Bullied	0.718 (0.293)	0.224	2.450	0.16

AQ: Autism Quotient; R^2^: 0.178, Adjusted R^2^: 0.164.

**Table 4:** Linear regression analysis performed using suicidal ideation as the dependent variable and AQ total score and being bullied as independent variable.

	B (S.E.)	Beta	t	*p*
constant	−0.182 (0.207)		−0.878	0.381
AQ total score	0.041 (0.011)	0.299	3.736	<0.001
Bullied	0.604 (0.218)	0.222	2.775	0.006

AQ: Autism Quotient; R^2^: 0.189, Adjusted R^2^: 0.178.

**Table 5:** Linear regression analysis performed using suicidal behavior as the dependent variable and AQ total score and being bullied as independent variable.

	B (S.E.)	Beta	t	*p*
constant	−0.149 (0.71)		−2.084	0.039
AQ total score	0.013 (0.004)	0.312	3.383	0.001
Bullied	0.092 (0.073)	0.116	1.254	0.212

AQ: Autism Quotient; R^2^: 0.137, Adjusted R^2^: 0.122.

**Table 6:** Comparison of MOOD-SR and AQ scores between subjects who showed only one criterion for the diagnosis of ASD who have or have not experienced bullying.

	BAP1	Bullied BAP1	t	*p*	Cohen’s d	C.I. 95%	
						Lower Bound	Upper Bound
MOOD-SR Suicidality							
Suic. id.	1.54 ± 1.66	1.87 ± 1.67	−0.541	0.593	1.666	−0.934	0.534
Suic. behave.	0.11 ± 0.33	0.08 ± 0.28	0.262	0.796	0.031	−0.738	0.963
Suic. tot.	2.00 ± 1.87	1.50 ± 1.83	0.613	0.547	1.850	−0.602	1.135
AQ							
Social Skill	3.12 ± 2.61	4.09 ± 2.80	−1.432	0.157	2.707	−0.851	0.138
Att. Switch.	4.34 ± 1.93	5.19 ± 1.51	−1.948	0.056	1.732	−0.983	0.012
Att. detail	4.31 ± 2.29	5.37 ± 2.27	−1.864	0.067	2.280	−0.961	0.033
Comm.	2.48 ± 1.73	3.50 ± 2.11	−2.087	0.041 *	1.932	−1.026	−0.021
Imagin.	2.81 ± 1.65	2.71 ± 1.59	0.251	0.803	1.626	−0.431	0.557
AQ tot.	16.98 ± 6.26	20.77 ± 6.51	−2.345	0.022 *	6.390	−1.102	−0.084
MOOD-SR							
Dep. Mood	29.19 ± 12.58	29.91 ± 11.04	−0.243	0.809	11.836	−0.551	0.430
Man. Mood	10.34 ± 5.02	12.03 ± 4.43	−1.425	0.159	4.735	−0.849	0.139
Dep. Energy	2.41 ± 2.30	4.28 ± 2.43	−3.174	0.002 *	2.363	−1.300	−0.281
Man. Energy	4.19 ± 2.75	5.19 ± 2.63	−1.485	0.143	2.693	−0.864	0.124
Dep. Cogn.	8.84 ± 5.84	11.56 ± 4.29	−2.122	0.038 *	5.124	−1.027	−0.030
Man. Cogn.	7.06 ± 4.65	8.66 ± 3.87	−1.490	0.141	4.279	−0.865	0.123
Rhythm.	8.19 ± 5.16	10.75 ± 4.30	−2.159	0.035 *	4.747	−1.037	−0.039
MOOD-SR tot.	70.22 ± 27.78	82.38 ± 20.81	−1.982	0.052	24.536	−0.991	0.004

BAP1: individuals endorsing only one autistic symptom; AQ: Autism Quotient; MOOD-SR: MOOD Spectrum-Self report; * significant for *p* < 0.05.

**Table 7:** Comparison of MOOD-SR and AQ scores between subjects with a clinical diagnosis of ASD who have or have not experienced bullying.

	ASD	Bullied ASD	t	*p*	Cohen’s d	C.I. 95%	
						Lower Bound	Upper Bound
MOOD-SR Suicidality							
Suic. id.	0.67 ± 1.15	2.00 ± 1.69	−1.291	0.215	1.633	−2.075	0.467
Suic. behave.	0.00 ± 0.00	0.58 ± 0.79	−2.548	0.027 *	0.729	−2.087	0.516
Suic. tot.	0.67 ± 1.15	2.50 ± 2.50	−1.864	0.101	2.348	−2.066	0.533
AQ							
Social Skill	5.86 ± 2.73	7.63 ± 2.04	−1.910	0.065	2.188	−1.659	0.050
Att. Switch.	7.28 ± 2.21	8.22 ± 1.58	−1.288	0.207	1.714	−1.384	0.300
Att. detail	6.71 ± 2.29	6.78 ± 1.99	−0.073	0.942	2.047	−0.862	0.801
Comm.	6.71 ± 2.29	7.44 ± 2.10	−0.806	0.426	2.137	−1.175	0.496
Imagination	4.71 ± 2.75	5.44 ± 1.99	−0.800	0.429	2.151	−1.172	0.499
AQ tot.	31.28 ± 10.11	35.52 ± 6.15	−1.412	0.168	7.067	−1.438	0.250
MOOD-SR							
Dep. Mood	61.00 ± 1.73	59.63 ± 9.82	0.236	0.816	9.247	−1.088	1.381
Mani. Mood	7.33 ± 1.15	10.44 ± 2.83	−3.195	0.013 *	2.686	−2.432	0.151
Dep. Energy	2.00 ± 1.00	5.69 ± 2.15	−2.859	0.011 *	2.050	−3.149	−0.406
Man. Energy	3.67 ± 3.79	5.25 ± 2.65	−0.897	0.382	2.804	−1.804	0.691
Dep. Cogn.	10.33 ± 2.08	14.88 ± 5.07	−1.499	0.152	4.817	−2.203	0.343
Man. Cogn.	6.00 ± 3.61	8.31 ± 3.42	−1.068	0.301	3.442	−1.916	0.591
Rhythm.	14.67 ± 3.05	14.63 ± 3.32	0.020	0.984	3.294	−1.221	1.246
MOOD-SR tot.	105.00 ± 6.56	118.81 ± 20.61	−1.126	0.276	19.491	−1.954	0.557

ASD: Autism Spectrum Disorder; * significant for *p* < 0.05.

## Data Availability

The original contributions presented in this study are included in the article. Further inquiries can be directed to the corresponding author.

## Abbreviations

The following abbreviations are used in this manuscript:

WHO	World Health Organization
ASD	Autism Spectrum Disorder
BAP	Broad Autism Phenotype
HCs	Healthy Controls
DSM	Diagnostic and Statistical Manual for Mental Disorders
AOUP	Azienda Ospedaliero Universitaria Pisana
AdAS Spectrum	Adult Autism Subthreshold Spectrum
AQ	Autism Spectrum Quotient
IQ	Intelligence Quotient
MOOD-SR	Mood Spectrum—Self Report
